# Surface effects on kinematics, kinetics and stiffness of habitual rearfoot strikers during running

**DOI:** 10.1371/journal.pone.0283323

**Published:** 2023-03-22

**Authors:** Wenxing Zhou, Lulu Yin, Jiayi Jiang, Yu Zhang, Cheng-pang Hsiao, Yiyang Chen, Shiwei Mo, Lin Wang

**Affiliations:** 1 Key Laboratory of Exercise and Health Sciences (Shanghai University of Sport), Ministry of Education, Shanghai, China; 2 School of Exercise and Health, Shanghai University of Sport, Shanghai, China; 3 Department of Physiotherapy, Monash University, Victoria, Australia; 4 Department of Rehabilitation Medicine, The Tenth People’s Hospital Affiliated to Tongji University, Shanghai, China; 5 Department of Kinesiology and Physical Activity, McGill University, Quebec, Canada; 6 Human Performance Laboratory, School of Physical Education, Shenzhen University, Shenzhen, Guangdong, China; Ningbo University, CHINA

## Abstract

The surface effects on running biomechanics have been greatly investigated. However, the effects on rearfoot strike runners remain unknown. The purpose of this study was to investigate the effects of surfaces on the running kinematics, kinetics, and lower-limb stiffness of habitual rearfoot strikers. Thirty healthy male runners were recruited to run at 3.3 ± 0.2 m/s on a customized runway covered with three different surfaces (artificial grass, synthetic rubber, or concrete), and their running kinematics, kinetics, and lower-limb stiffness were compared. Differences among the three surfaces were examined using statistical parametric mapping and one-way repeated-measure analysis of variance. There were no statistical differences in the lower-limb joint motion, vertical ground reaction force (GRF), loading rates, and lower-limb stiffness when running on the three surfaces. The braking force (17%–36% of the stance phase) and mediolateral GRF were decreased when running on concrete surface compared with running on the other two surfaces. The moments of ankle joint in all three plane movement and frontal plane hip and knee joints were increased when running on concrete surface. Therefore, habitual rearfoot strikers may expose to a higher risk of running-related overuse injuries when running on a harder surface.

## Introduction

Running is one of the most popular physical activities worldwide and usually performed on different road surfaces. Different running surfaces were speculated to be associated with overuse injuries. However, all evidence to support this statement is circumstantial. Lorimer and Hume [1] concluded that running on roads with soft surfaces or low surface stiffness (i.e., sand) would increase Achilles injury risk. McMahon and Greene [2] stated that a “tuned” surface can alleviate overuse injuries, whereas Van Mechelen [3] claimed no significant association between overuse injuries and hard surfaces. It remains inconclusive about the relationship between road surfaces and overuse injury risk.

Synthetic rubber, concrete, and asphalt surfaces are the three most common road surfaces for distance runners [4, 5]. Artificial grass surface is also popular among recreational runners [6, 7]. Different road surfaces may differ in flatness, stiffness, and/or elasticity, thereby altering running biomechanics. Regarding running kinematics, Hardin et al. [8] reported that increased surface stiffness led to adaptive changes in lower-limb kinematics (i.e., decreased hip and knee flexion at initial contact, reduced maximal hip flexion). In addtion, rearfoot strikers runners exhibit greater foot pronations and a more plantarflexed foot when running on a harder road surface (i.e., concrete or asphalt) than on a softer road surface (i.e., grass or synthetic rubber) [9, 10]. Regarding running kinetics, the average vertical loading rate (AVLR, defined as the average slope of the line between 20% and 80% of the vertical impact peak) [11] and the ankle joint moments were found to be lower and knee joint moments were greater when running on a softer road surface than on a harder road surface [10, 12]; the lower-limb stiffness was found to decrease with the road surface stiffness [13, 14]. Furthermore, running on stiffer surfaces (i.e., synthetic track or concrete surface) are accosiated with lower plantar pressure, vertical impact and horizontal braking forces compared with running on softer surfaces (i.e., natural grass) [4, 5, 9, 15]. However, inconsistent results regarding the aforementioned kinetic variables were also largely reported in previous studies [16, 17]. For example, Fu et al. [17] reported no differences in plantar pressure among concrete, synthetic track, or natural grass surface. Shen et al. [16] observed no differences in vertical impact peak between asphalt road and plastic track. Such inconsistency may be attributed to many confounding factors, such as running experience [18], running speed [9], and/or footwear [10, 19]. Thus, in efforts to further understand which the more proper running surfaces for runners, it is critical to better control other risk factors.

Additionally, runners may alter their foot strike patterns and landing mechanics when running on roads with different surface stiffnesses [19], thereby changing their running biomechanics. However, previous studies did not categorize runners according to their foot strike patterns when examining the effects of surfaces on their running biomechanics [18, 20, 21]. One study [22] investigated the effects of overground surfaces on running biomechanics of non-rearfoot strike runners. However, the effects of overground surfaces on running biomechanics remain largely unknown in rearfoot strike runners.

Therefore, this study aimed to compare the running biomechanics of habitual rearfoot strikers on three different overground surfaces (artificial grass, synthetic rubber, and concrete). It was hypothesized that habitual rearfoot strikers would present lower ground reaction forces (GRFs), vertical loading rates, and lower-limb joint moments and stiffness when running on a softer surface (artificial grass or synthetic rubber) than on a harder surface (concrete).

## Methods

### Subjects

Thirty healthy male runners (age = 24.3 ± 2.4 years; body height = 1.72 ± 0.06 m; body mass = 67.9 ± 10.5 kg) voluntarily participated in this study. All the runners are self-reported to be right-leg dominant. We confirmed it by asking them to kick a ball [23] and all of them preferred to kick the ball using their right foot. All the runners are habitual rearfoot strikers and have been running regularly for 3.0 ± 1.8 years (≥10 km/week) on overground surfaces (i.e., grass, synthetic rubber, or concrete). Each runner’s foot strike pattern was determined by asking them to run at their comfortable speeds in Pedar® insoles (Novel, Munich, Germany). Their rearfoot strike pattern was confirmed because the location of the center of pressure at initial contact was consistently between 0–33% of foot length [24]. All the procedures of the current study met the current ethical standards for human research and was approved by the ethics committee of Shanghai University of Sport (#2018076). All the runners signed a written consent before data collection.

An *a priori* sample size was estimated through G*Power 3.1 using previous lower-limb kinematic and kinetic data [8, 17]. Twenty-one participants were deemed to sufficient to obtain a desired power of 80% at α = 0.05.

### Procedures

The experiment was conducted in a biomechanical laboratory. The running movements were captured using a 10-camera motion capture system (Vicon, Oxford, UK; 200 Hz), and the GRFs were recorded using two force plates (90 cm × 60 cm; Kistler 9287 C, Winterthur, Switzerland; 1000 HZ). The force plates were embedded in the middle of the runway, at around 8 meters from the starting line. Detailed information about the experimental setup and the methods of data acquisition can be found in our previous publication [22]. A total of 23 retroreflective markers were attached to the runner’s pelvis (sacrum, the superior border of the iliac crests, and the anterior superior iliac spines) and lower limbs (the greater trochanter, the medial and lateral femoral epicondyles, the medial and lateral malleoli, the first and fifth metatarsal heads, the second toe tip, and the heel). Foot markers were placed on the shoes at the corresponding anatomical locations. An experienced musculoskeletal physiotherapist positioned the markers in all conditions. During data acquisition, all the runners were instructed to run at 3.3 ± 0.2 m/s on a 15-m runway. The entire length of the runway was covered with artificial grass (20 mm, close to the layer of the overused artificial turf court), synthetic rubber (20 mm), or concrete surface (10 mm). In order to increase friction, polyvinyl chloride mattress (1.6 mm) was placed between the runway, force plate surface and the covered materials. The surface over the force plates was isolated from the rest of the runway by a small gap (5 mm). The surface hardness was measured by a ball drop test adopted from the study by Fu et al [17]. Specifically, a basketball (size 7# with an air pressure of 0.06 MPa) was vertically dropped from a height of 2 m on each surface. The bounce height of the ball was recorded, and the coefficient of restitution was calculated for each surface using the surface thickness normalized bounce height and the drop height [25]. The coefficient of restitution for the artificial grass, synthetic rubber and concrete surfaces were respectively 0.29 ± 0.001, 0.32 ± 0.002 and 0.42 ± 0.010. The hardest surface was concrete, followed by synthetic rubber and artificial grass.

They were initially instructed to familiarize themselves with the entire procedure and ensure that they are running at the target speed through practice. To gain comparable results among different surfaces, the running speed controlled at 3.3 m/s, which was widely recommended in previous studies [17, 26], and also close to the comfortable speed reported by our participants. During data collection, the two photocell sensors of the timing system were positioned 3 m apart along the runway for monitoring the speed when the participant ran through the force plate area. The runners were provided with the same running shoes (ASICS SORTIEMAGIC RP 4 TMM467-0790, Japan; European size 41 to 43), and the testing order was randomized. For each surface condition, the running test was finished if 5 successful running trials were obtained. Overall, each runner performed 5–10 trials for each surface condition. To avoid fatigue accumulation, each runner was allowed to rest between trials if required and was required to rest at least 5 minutes between surface conditions.

### Data analysis

Five successful running trials for each surface condition were selected for analysis. A sucessful running trial was defined as follows: (i) the entire right foot striked on one of the two force plates, and (ii) the running speed was within 3.3 ± 0.2 m/s.

The Visual 3D software (C-Motion Inc., Rockville, MD, USA) was used to compute the kinematics (joint angles) and the kinetics (GRFs and joint moments) and identify the stance phase (from initial contact to toe off). Initially, the raw marker trajectories and GRFs were respectively low-pass filtered at 10 and 50 Hz with a fourth-order, zero lag Butterworth filter [27]. Initial contact and toe-off events were determined using a threshold of 20 N [28]. Joint angles for the right hip, knee and ankle joints were calculated in sagittal, frontal and transverse planes. The corresponding joint moments were computed through an inverse dynamic approach and were expressed as external moments. The GRFs and joint moments were normalized by the runner’s body mass. All data (joint angles, joint moments, GRFs) were time normalized (101 samples) to express them as a percentage of the stance phase from 0 to 100%.

To evaluate the landing impact, the AVLR and the instantaneous vertical loading rate (IVLR) were respectively obtained by computing the average and steepest slopes of the line through the 20% and 80% points of the vertical impact peak in the raw GRF data [11]. The lower-limb stiffness was quantified using the leg stiffness (*k*_*leg*_), vertical stiffness (*k*_*vert*_), and joint stiffness (*k*_*joint*_), which are respectively calculated using the following equations:

$$k_{leg}=\frac{vGRF_{max}}{\Delta L},$$ (1)

$$k_{vert}=\frac{vGRF_{max}}{\Delta y},$$ (2)

$$k_{joint}=\frac{\Delta M}{\Delta \theta }$$ (3)

where *k*_*leg*_ is the ratio of the maximum vertical GRF (*vGRF*_*max*_) to the leg spring compression (Δ*L*) [29], *k*_*vert*_ is the ratio of *vGRF*_*max*_ to the vertical displacement of the center of mass Δ*y* [30], and *k*_*joint*_ is the ratio of the total change in joint moment (Δ*M*) to the corresponding change in joint angular displacement (Δ*θ*) [31], which was computed for the knee and ankle joints. Leg spring compression (Δ*L*) is the vertical variation of the leg length, which is $\Delta L=L_{0}-\sqrt{L_{0}^{2}-{\left(\frac{1}{2}vt_{c}\right)}^{2}}+\Delta y$, where *L*_*0*_ is the leg length from the greater trochanter to the prominence of the lateral malleolusis, *v* is the running velocity, and *t*_*c*_ is the stance time. The calculations and the post-processing were performed in MATLAB (version R2021b, MathWorks Inc., Natick, MA).

### Statistical analysis

The mean lower-limb joint angles, moments, stiffness, and GRF values were calculated by averaging the relevant data from the five successful trials for each surface condition for each runner. Statistical analysis was performed using SPSS (version 26.0, SPSS Inc., Chicago, IL, USA) and MATLAB (The Mathworks Inc., USA). One-way repeated-measure analysis of variance (ANOVA) was performed to examine the differences of the discrete variables (AVLR, IVLR, *k*_*leg*_, *k*_*vert*_, *k*_*joint*_). If the significance was indicated, then post-hoc pairwise comparisons with Bonferroni correction were performed. Further, statistical parametric mapping (SPM) of one-way repeated-measures ANOVA was employed to compare the GRF, lower-limb joint angle and moment curves of the three surface conditions using an open-source code (www.spm1D.org) in MATLAB. If the significance values were indicated, then the SPM of the paired *t* test with Bonferroni correction was performed. This approach is an efficacious supplement to discrete analyses, as data of the entire gait stance phase are compared [32]. The significance level was set to α = 0.05.

## Results

### Running kinematics

**Fig 1:**
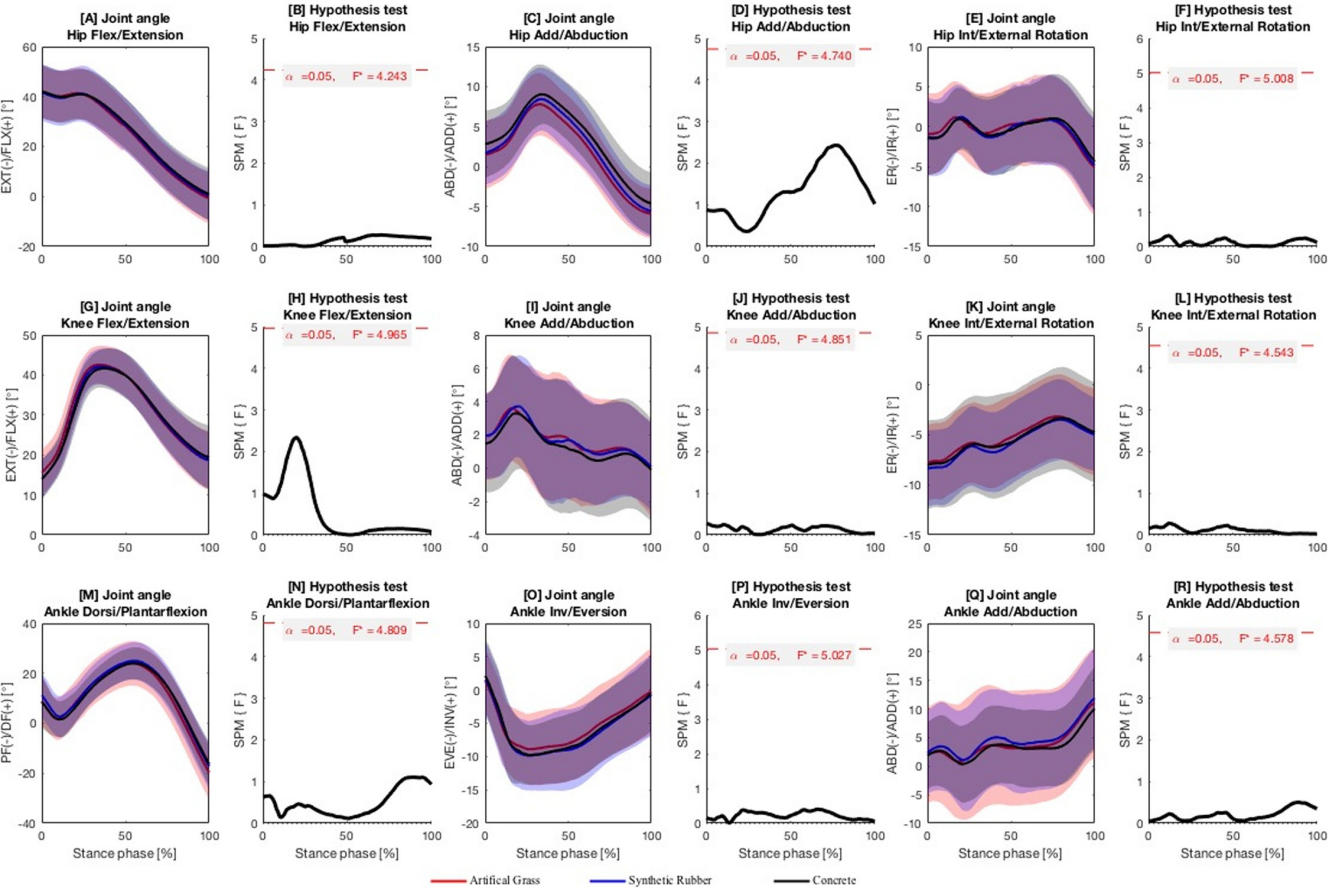
Lower extremity joint angles. Ensemble time series of 3D lower-limb joint angles and the corresponding results of statistical parametric mapping analysis (hip joint angle: A–F; knee joint angle: G–L; ankle joint angle: M–R) across participants during running on artificial grass, synthetic rubber and concrete surfaces. Areas of significant differences are marked in shaded grey. F* = critical threshold.

The differences in the hip, knee, and ankle joint angles of the three planes when running on artificial grass, synthetic rubber, and concrete surfaces were not significant (Fig 1; *p*s > 0.05).

### Running kinetics

**Discrete variables of loading rate and lower-limb stiffness.** No statistical differences were observed in the vertical loading rates (AVLR and IVLR) and lower-limb stiffnesses (*k*_*leg*_, *k*_*vert*_, *k*_*joint*_) among the three surfaces (Table 1; *p*s ≥ 0.14).

**Table 1:** Comparison of vertical loading rates and lower-limb stiffness (mean ± standard deviation) during running on artificial grass, concrete, or synthetic rubber surface.

	Variables	Artificial grass	Synthetic rubber	Concrete	One-way analysis of variance		
					F value	*p* value	* $\eta_{p}^{2}$ *
Vertical loading rates	AVLR (BW/s)	147.18±65.33	154.02±72.81	141.22±68.55	1.28	0.29	0.04
	IVLR (BW/s)	207.89±90.66	215.70±91.76	204.63±88.29	0.63	0.53	0.02
Lower-limb stiffness	*K*_*leg*_ (KN/m)	8.56±1.78	8.65±1.70	8.85±1.88	0.79	0.43	0.03
	*k*_*vert*_ (KN/m)	19.96±3.68	19.96±3.47	20.19±3.60	0.14	0.87	0.005
	Knee *k*_*joint*_ (Nm/kg/°)	0.04±0.01	0.04±0.01	0.03±0.01	2.05	0.14	0.07
	Ankle *k*_*joint*_ (Nm/kg/°)	0.05±0.01	0.05±0.01	0.06±0.02	1.26	0.28	0.04

VALR: vertical average loading rate; VILR: vertical instantaneous loading rate; *K*_*leg*_: leg stiffness; *k*_*vert*_: vertical stiffness;*k*_*joint*_: joint stiffness

**Fig 2:**
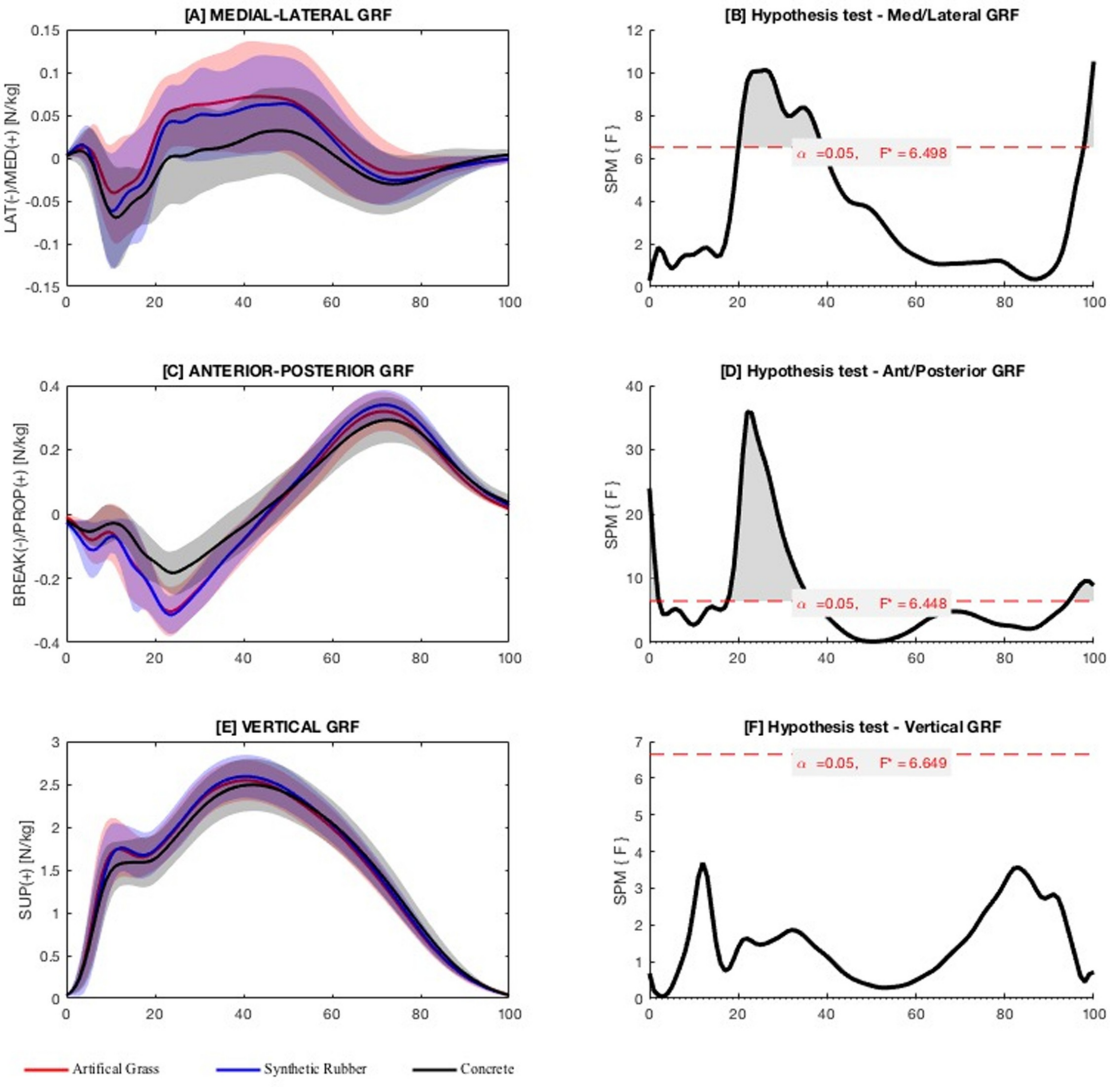
Ground reaction forces. Ensemble time series of 3D GRF forces (A, C, E) and the corresponding results of statistical parametric mapping analysis (B, D, F) across participants during running on artificial grass, synthetic rubber and concrete surfaces. Areas of significant differences are marked in shaded grey. F* = critical threshold.

**Continuous variables of ground reaction force.** The difference in vertical GRF was not significant when running on the three surfaces (Fig 2). However, significant differences in the anteroposterior GRF (braking and propulsion forces) were observed in the following ranges: 0% to 2% (*F*_2,87_ = 6.45, *p* = 0.04), 17% to 36% (*F*_2,87_ = 6.45, *p* < 0.001), and 94% to 100% (*F*_2,87_ = 6.45, *p* = 0.019). Specifically, the braking force (0%–2%) when running on artifical grass surface were significantly greater than synthetic rubber (*p* = 0.015) or concrete surface (*p* = 0.017), whereas the propulsion force (94%–100%) when running on artificial grass was lower than synthetic rubber (*p* = 0.004) or concrete surface (*p* = 0.001); the braking force (17%–36%) was significantly greater when running on artificial grass (*p* < 0.001) or synthetic rubber (*p* < 0.001) than concrete surface. Significant differences were also detected in the mediolateral GRF (20%–38%, *F*_2,87_ = 6.49, *p* < 0.001; 98%–100%, *F*_2,87_ = 6.49, *p* = 0.042). However, the significant differences were only observed between the artifical grass and concrete surfaces (*p* < 0.001 and 0.016, respectively).

**Fig 3:**
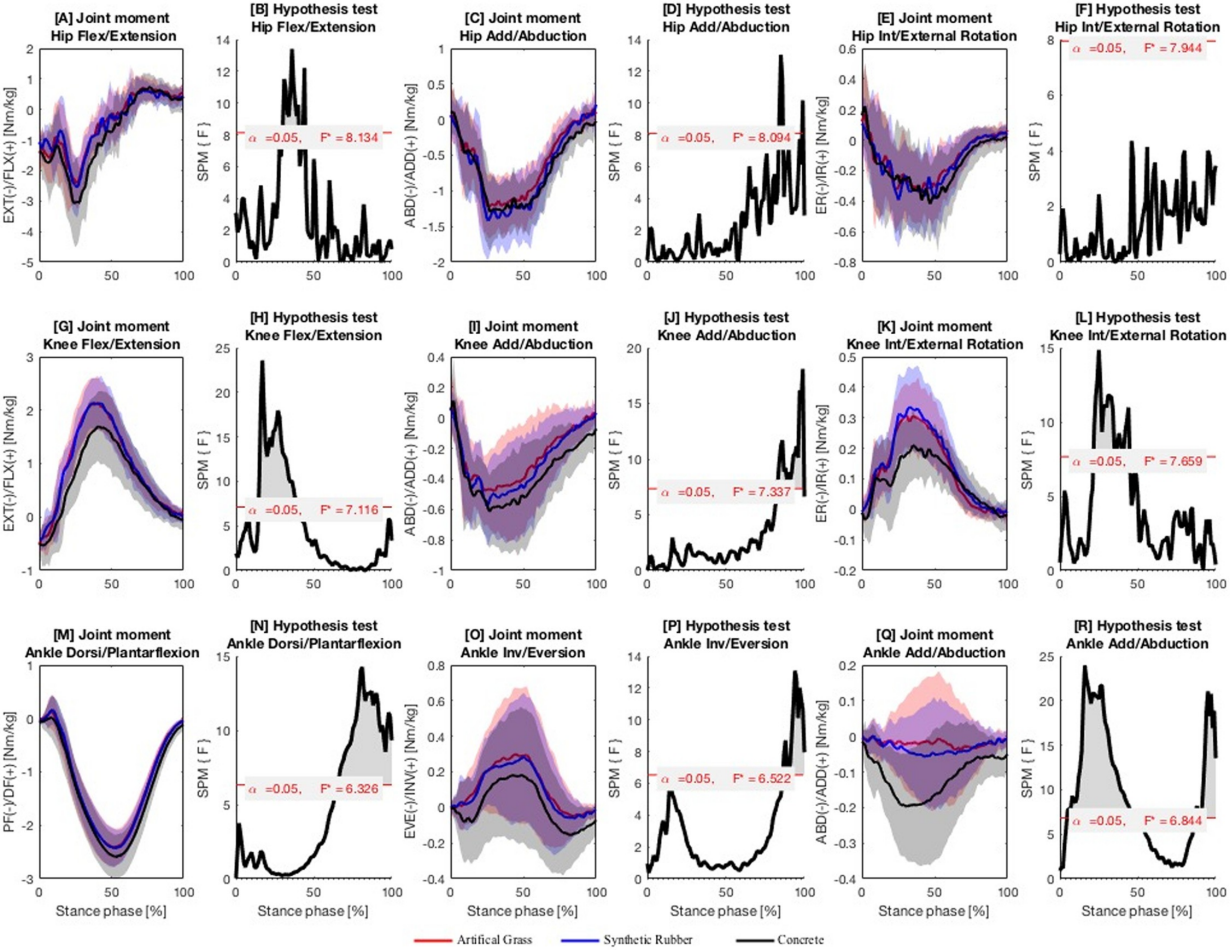
Lower extremity joint moments. Ensemble time series of 3D joint moments and the corresponding results of statistical parametric mapping analysis (hip joint moment: A–F; knee joint moment: G–L; ankle joint moment: M–R) across participants during running on artificial grass, synthetic rubber and concrete surfaces. Areas of significant differences are marked in shaded grey. F* = critical threshold.

**Continuous variables of lower-limb joint moment.** Significant differences in lower-limb joint moments were detected when running on the three surfaces (Fig 3; *p* < 0.05). Regarding the hip joint, the differences in the hip flex/extension moment at the end of the braking phase (30%–40%, *F*_2,87_ = 8.13, *p* < 0.001; 43%–45%, *F*_2,87_ = 8.13, *p* = 0.007) and the hip abd/adduction moment at the end of the propulsion phase (84%–86%, *F*_2,87_ = 8.09, *p* < 0.001; 98%–99%, *F*_2,87_ = 8.09, *p* = 0.035) were significant. Post-hoc pairwise comparison showed that the hip extension and abduction moments were lower when running on artificial grass (*p*s ≤ 0.016) or synthetic rubber (*p*s ≤ 0.014) than on concrete surface.

Regarding the knee joint (Fig 3), significant differences were observed in all three planes: knee flex/extension moment (14%–39%, *F*_2,87_ = 7.12, *p* < 0.001), knee abd/adduction moment (83%–100%, *F*_2,87_ = 7.34, *p* < 0.001), and knee int/external rotation moment (22%–44%, *F*_2,87_ = 7.66, *p* < 0.001). Post-hoc pairwise comparisons indicated that the knee flexion and internal rotation moments when running on artificial grass (*p*s ≤ 0.004) or synthetic rubber (*p*s ≤ 0.004) were greater whereas the knee abduction moment (*p* ≤ 0.005 and ≤ 0.016, respectively) was significantly lower than on concrete surface.

The ankle joint moments present signficant differences in all three planes when running on the three surfaces (Fig 3): ankle dorsi/plantarflexion moment (65%–100%, *F*_2,87_ = 6.33, *p* < 0.001), ankle inv/eversion moment (86%–100%, *F*_2,87_ = 6.52, p < 0.032), and ankle abd/adduction moment (5%–47%, *F*_2,87_ = 6.84, *p* < 0.001; 86%–100% (*F*_2,87_ = 6.84, *p* < 0.037)). Post-hoc pairwise comparisons showed that the ankle plantarflexion, eversion, and abduction moments when running on artificial grass (*ps* ≤ 0.013) or synthetic rubber (*ps* ≤ 0.015) were lower than those when running on concrete surface.

## Discussion

This study compared the running biomechanics and lower-limb stiffness of habitual rearfoot strike runners on a runway covered with three different surfaces (artificial grass, synthetic rubber, or concrete). The lower-limb joint moments were lower when running on artificial grass or synthetic rubber, except for the knee flexion and internal rotation moments. This finding partially supports our initial hypothesis. The braking force (17%–36% of the stance phase) and mediolateral GRF were significantly greater when running on a soft surface (artificial grass or synthetic rubber) than on a hard surface, and the lower-limb stiffness, the vertical GRF, the loading rates, and the lower-limb joint motion showed no significant differences among the three surfaces, which are inconsistent with our original hypothesis.

### Running kinematics

The runners did not show any differences in lower-limb joint motion when running on the three surfaces. However, previous studies reported that runners were observed to modify their running kinematics according to the road stiffness. Shen et al. [16] reported a lower peak ankle dorsiflexion and hip flexion for the plastic track (softer) than the asphalt road (harder); Pinnington et al. [33] indicated an increased peak knee flexion angle when running on a soft, dry sand surface compared with that when running on a firm, wooden floor surface. In our study, the runners did not adjust their running postures, which may be due to the insufficient differences in road stiffness among the three artificial runways. In the current study, we added different materials (artificial grass, synthetic rubber, concrete) on the top of the original pavement without making more changes to its internal structure, which may explain the unchanged running kinematics.

### Running kinetics

Theoretically, running on different road surfaces would generate different landing impacts, especially in the vertical direction. However, in the current study, we demonstrated no differences in the vertical GRF and loading rates when running on the three overground surfaces. Consistent to our finding, Yamin et al. [7] concluded that GRF was not in correlation with the surface hardness when running in minimally or heeled shoe. Dixon et al. [12] reported no differences in peak impact force among conventional asphalt surface, rubber-modified asphalt surface and acrylic sports surface. Yet, discrete parameters (i.e., peak GRF by Yamin et al. and peak impact force by Dixon et al.) were compared in their study. Unlike the two aforementioned studies, the current study compared the entire vertical GRF curves as well as loading rates (AVLR and IVLR). Thus, our findings may provide further evidence to support that the vertical GRF was typically not influenced by the change in surface [7, 12].

Researchers still have different views regarding the surface effects on the vertical GRF and loading rates. Some studies [12, 34–36] demonstrated that surface stiffness did not affect the vertical GRF during running. Other studies reported that the vertical GRF changed with surfaces stiffness [7, 14]. When the plantar pressure values instead of the vertical GRF values were compared, current evidence suggests that softer surfaces yield lower [4, 5, 15] or similar [17] magnitudes of peak plantar pressure values compared to harder surfaces. Shen et al. [16] found the AVLR was not different when running on asphalt road or plastic track, whereas Dixon et al. [12] reported greater vertical loading rates when running on hard surfaces. The surface effect on landing impact during running seems remain inconclusive. Therefore, besides surface stiffness, further studies are required to unveil the potential relationship by considering many factors, such as surface structures, runners’ footstrike patterns, shoe conditions, and quantification methods.

We did not observe any difference in the lower-limb stiffness measurements (e.g., *k*_*leg*_, *k*_*vert*_, or *k*_*joint*_) when the habitual rearfoot strikers ran on the three different surfaces. These results can be explained by the unchanged lower-limb joint kinematics and vertical GRF. Inconsistent to our study, scholars demonstrated that runners subconsciously adjusted their lower-limb stiffness based on the perception of different surface hardness [37]. Indeed, runners were previously found to increase their leg stiffness and decrease the knee and ankle joint stiffness when running on softer surfaces [6, 13, 14]. However, in the current study, as the running speed was close to participant’s comfortable speeds, they did not need to modify their running postures to maintain performance.

The braking force at initial contact (0%–2% of stance phase) was significantly lower when running on artificial grass compared with running on synthetic rubber or concrete surface but became greater during the middle of the braking stage (17%–36% of stance phase). This phenomenon indicates the different braking mechanisms of habitual rearfoot strikers when running on different surfaces. Particularly, the forward acceleration was reduced slowly when running on artificial grass instead of decelerating suddenly like they did during running on synthetic rubber or concrete surface. In addition, the propulsion force at the end (94%–100%) of the stance phase was lower when running on artificial grass than that when running on the other surfaces. The greater propulsion force means that the runners could push off the ground more forcefully when running on synthetic rubber or concrete surface than on artificial grass. Horizontal GRF was rarely investigated. Only one study by Karamanidis et al. [35] compared anteroposterior GRF when running on three surfaces with different compliance. However, they reported no differences in the braking and propulsion forces when running on road surfaces with different stiffness. It seems that, besides the surface stiffness, other features of the running road (i.e., surface coefficient of friction) may also play a role, which should be investigated in the future.

Regarding the mediolateral GRF, the medial force in the early stance phase and the lateral force during the late stance phase were greater when running on artificial grass than concrete surface. The mediolateral GRF allows the quantification of the path of the center of mass (COM) in the frontal plane [38] and is related to the gait stability during running. The excessive transition of COM from medial to lateral (or lateral to medial) during running requires a great force to stabilize the runners’ posture in the frontal plane [38]. Accordingly, the dynamic postural stability was lower when running on softer surfaces (e.g., woodchip trail) [39]. Therefore, the mediolateral GRF of our habitual rearfoot strikers running on artificial grass were high.

Although we did not observe any differences in lower-limb kinematics, stiffness and vertical GRF when our habitual rearfoot strikers ran on the three different surfaces, their ankle joint moments were greater when running on concrete surface than the other two surfaces. The hip extension, hip abduction and knee abduction moments were also greater when running on the harder surface. Generally, the external joint moments give a net estimate of the loading imposed on the biological structures surrounding a joint, such as muscles, tendons and ligaments. In our study, increased ankle joint moments mostly occurred during the propulsion phase (65%-100%) when the foot pushed the body forward, which increases more stress for plantar flexors and subtalar joint invertors when running on the harder surface. Increased knee and hip joint moments occurred at the end of propulsion phase for the frontal plane movement when running on the harder surface, which may result in greater retropatellar stress through greater contributions from the vastus lateralis, extensions of the iliotibial band, or both [40]. In addition, previous studies have attempted to decrease frontal hip or knee joint moments through training program to reduce running-related overuse injuries [41]. Willwacher et al. [10] recently observed that running barefoot on harder surfaces increased the ankle joint moments and decreased knee joint moments. They explained it to a more plantar flexed footstrike patterns, which are usually associated with barefoot running or running on harder surfaces. Unlike our study, their subjects adopted a more plantarflexed footstrike behavior. In a word, our findings may demonstrate that the habitual rearfoot strikers may continuously expose to a higher risk of running-related overuse injuries when running on the harder surface.

Two limitations should be noted. First, only male runners were recruited in this study, which may prevent the generalization our findings because of gender differences in shock attenuation when running on different surfaces [42]. Second, the running tests were conducted on a short constructed laboratory runway (15 m), and they can run relatively limited steps before landing on the force plates. Meantime, relative short time was provided to each runner to familiraze themselves with the three new running surfaces. It is necessary to conduct an outdoor running test to further investigate the surface effects on running biomechanics.

### Conclusion

Habitual rearfoot strike runners showed lower braking force (17%–36% of the stance phase) and mediolateral GRF and continuously displayed greater joint moments for ankle joint in all three plane movement and frontal plane hip and knee joints when running on a hard road surface (concrete vs. artifical grass or synthetic rubber). Meanwhile, they could maintain their lower-limb joint motion, loading rates, vertical GRF, and stiffness at a relatively constant level when running regardless of the road surface. Overall, habitual rearfoot strikers may expose to a higher risk of running-related overuse injuries when running on a harder surface.

## Supporting information

S1 Data Raw data of main figures used in this study. (ZIP)
